# Stable P3HT: amorphous non-fullerene solar cells with a high open-circuit voltage of 1 V and efficiency of 4%†

**DOI:** 10.1039/c9ra03188j

**Published:** 2019-07-03

**Authors:** HyunKyung Lee, Sora Oh, Chang Eun Song, Hang Ken Lee, Sang Kyu Lee, Won Suk Shin, Won-Wook So, Sang-Jin Moon, Jong-Cheol Lee

**Affiliations:** Advanced Materials Division, Korea Research Institute of Chemical Technology (KRICT) 141 Gajeong-ro, Yuseong Daejeon 34114 Republic of Korea leejc@krict.re.kr; Energy Materials Research Center, Korea Research Institute of Chemical Technology (KRICT) 141 Gajeong-ro, Yuseong Daejeon 34114 Republic of Korea songce@krict.re.kr; Advanced Materials and Chemical Engineering, University of Science and Technology (UST) 217 Gajeongro, Yuseong Daejeon 34113 Republic of Korea; KU-KRICT Collaborative Research Center, Division of Display and Semiconductor Physics, Department of Advanced Materials Chemistry, Korea University 2511 Sejong-ro Sejong 30019 Republic of Korea

## Abstract

A non-fullerene small molecule acceptor, SF-HR composed of 3D-shaped spirobifluorene and hexyl rhodanine, was synthesized for use in bulk heterojunction organic solar cells (OSCs). It possesses harmonious molecular aggregation between the donor and acceptor, due to the interesting diagonal molecular shape of SF-HR. Furthermore, the energy level of SF-HR matches well with that of the donor polymer, poly(3-hexyl thiophene) (P3HT) in this system which can affect efficient charge transfer and transport properties. As a result, OSCs made from a P3HT:SF-HR photoactive layer exhibited a power conversion efficiency rate of 4.01% with a high *V*_OC_ of 1.00 V, a *J*_SC_ value of 8.23 mA cm^−2^, and a FF value of 49%. Moreover, the P3HT:SF-HR film showed superior thermal and photo-stability to P3HT:PC_71_BM. These results indicate that SF-HR is specialized as a non-fullerene acceptor for use in high-performance OSCs.

## Abstract 2

A 3D-shaped SF-HR was designed and synthesized for use in non-fullerene organic solar cells. Owing to the aligned energy levels, the P3HT:SF-HR system exhibited a high efficiency of 4.01% with good thermal stability and photostability.

## Graphical abstract

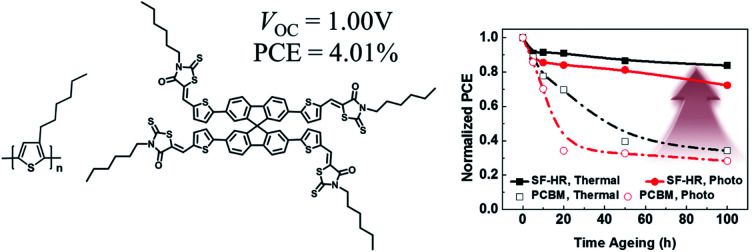

## 1. Introduction

Fullerene-based organic solar cells (OSCs) have received tremendous amounts of attention and have been significantly developed over the past decade.^1–8^ Fullerene derivatives such as [6,6]-phenyl-C_*x*_-butyric acid methyl ester (*x* = 61 and 71) (PC_*x*_BM) are employed as electron acceptors and have distinct features, such as low reorganization energy levels, long exciton lifetimes in a charge-separated state, and highly distributed electron mobility.^9–12^ However, due to the limitations of the absorption properties and energy level control, non-fullerene acceptors (NFAs) are being investigated widely and have caught up with the power conversion efficiency (PCE) based on those of fullerene-based OSCs. Especially, NFAs incorporated with an indacenodithieno[3,2-*b*]thiophene (IDTT) based coplanar backbone skeleton unit OSCs have shown PCEs of more than 14%.^13,14^ A planar backbone consisting of indaceno[1,2-*b*:5,6-*b*0]dithiophene (IDT) or IDTT-based small-molecule acceptors may lead to suitable lowest unoccupied molecular orbital (LUMO) and highest occupied molecular orbital (HOMO) energy levels to thus facilitate hole and electron transfers between the donor and acceptor components.^15,16^ In particular, coplanar backbones can establish π–π stacking effectively to accelerate mobile carrier transport, leading to acceptor aggregation. However, at times it could exceed the ideal exciton diffusion length scale due to the large planar structures and strong aggregates, leading to large domains in photoactive films that degrade the OSC performances. As an alternative strategy to reduce the self-aggregation tendency, a three-dimensional (3D) molecular geometry of an electron acceptor is employed for OSCs.^17–20^ 3D-structured NFAs are among the most promising candidates to inhibit the formation of large acceptor crystalline aggregation and excessively large domains in OSCs.^21,22^ A complete amorphous acceptor region to create 3D interconnecting charge transport networks with sufficient electron mobility has also been proposed. Thus, this strategy of a balanced 3D molecular acceptor with a polymer donor can lead to the successful enhancement of the photovoltaic performance. For example, a tetraphenylethylene-core-based perylene-diimide (PDI) NFA blended with the polymer donor PBDTT-F-TT demonstrated a PCE as high as 5.53% with a small domain size of ∼20 nm.^23^ Moreover, SF(DPPB)4, in which a spirobifluorene (SF) core is interlinked with four benzene end-capped diketopyrrolopyrrole (DPP) arms, provided the best PCE of 5.16%.^24^ It is expected that 3D-based NFAs have the potential to lead to the development of better photovoltaic performance outcomes.

To encourage a higher PCE value, not only a well-established molecular structure but also suitable energy level alignment would be important. The energy levels of the NFA should be aligned to the donor energy level to maximize the open-circuit voltage (*V*_OC_) and minimize the energy loss while maintaining efficient electron transfer. A number of reported NFAs have shown high *V*_OC_ values (0.8–1.2 V) through the optimization of these energy levels in photoactive materials, giving them a significant advantage over fullerene acceptors.^25,26^ These advances can promote the photovoltaic parameter, especially *V*_OC_, and facilitate electron transfer from the donor to the acceptor and hole transfer from the acceptor to the donor. With the management of the basic requirements of these specific properties, highly efficient photovoltaic performance even higher than that of fullerene acceptors can be expected.

Herein, we report a high-performance NFA OSC with the representative and promising poly(3-hexyl thiophene) (P3HT) donor and a spiro-based SF-HR acceptor. The acceptor, SF-HR, as used in this study is modified from SF-OR using SF as the center core unit linked with four 3-octylrhodanine arms, as reported by Qiu *et al.*^27^ OSCs based on a SF-OR acceptor blended with P3HT showed a maximum PCE of 4.66%, higher than those of PCBM-based devices. SF-HR is designed with thiophene linkers and 3-hexylrhodanine end cappers, thus providing more optimized solubility and minimizing the steric hindrance of the alkyl-chain between the acceptor molecules. Interestingly, with modification of the structure, an impressively high *V*_OC_ of 1.00 V was obtained, leading to a PCE of 4.01%. This high performance was achieved due to the good alignment energy level and harmonious molecular aggregation between the P3HT and SF-HR. Furthermore, the P3HT:SF-HR devices exhibited remarkable thermal and photo stability compared to PCBM-based OSCs. With the combination of the low-bandgap PTB7-Th donor polymer and SF-HR NFA, however, a poor PCE of 0.13% was obtained, attributable to the lack of energy level matching. Interestingly, SF-HR has been blended with PC_71_BM to understand the electron-donating or accepting characteristics of the SF-HR component. These devices showed a poor PCE of 0.02%, indicating that the SF-HR is a unipolar organic semiconductor as an electron acceptor. Consequently, these results demonstrate that SF-HR is a promising small-molecule NFA when combining P3HT.

## 2. Results and discussion

### 2.1. Molecular characterization

**Fig. 1:**
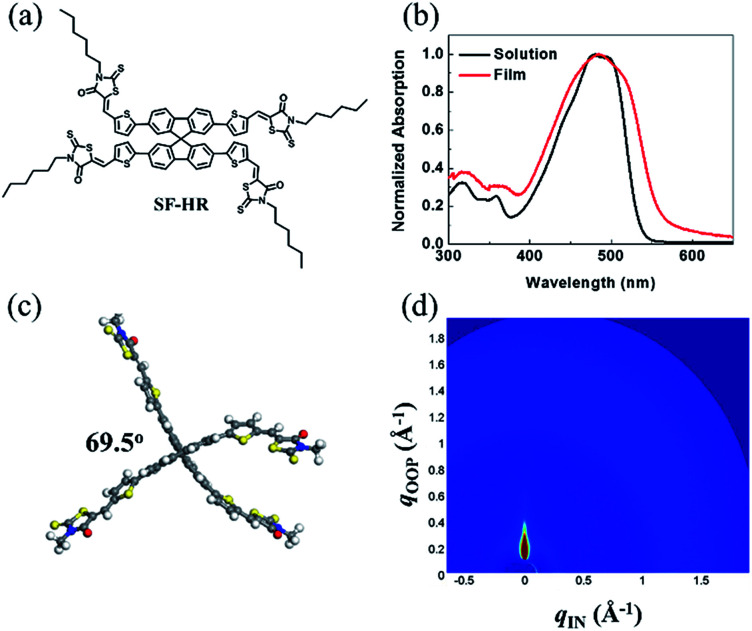
(a) Chemical structure of SF-HR. (b) UV-vis absorption spectra of SF-HR solution and film. (c) Ground-state geometry of SF-HR calculated by DFT method. (d) 2D GIWAXS image of neat SF-HR.

Fig. 1 shows the chemical structure of the NFA SF-HR used in the study. The detailed synthetic route and the material analysis of the SF-HR are described in the ESI.† The spiro-based NFA has good solubility in common organic solvents such as chloroform and chlorobenzene to create bulk heterojunction (BHJ) OSCs with established polymer donors. The thermal properties were explored by a thermogravimetric analysis (TGA) and differential scanning calorimetry (DSC) (see Fig. S1†). The thermal decomposition behavior of the SF-HR provides evidence of excellent thermal stability with a decomposition temperature (*T*_d_, 5% weight loss) of 330 °C in a nitrogen atmosphere. In addition, the DSC spectrum indicates that there is no phase transition peak ascribed to melting or crystallinity between 50 °C and 300 °C, suggesting that SF-HR has amorphous characteristics. Fig. 1b shows the UV-vis absorption spectra of SF-HR as a solution and a thin film. The absorption onset of the SF-HR film appears at 550 nm and is red-shifted by 20 nm from that in the solution, which may reflect self-organization behavior in a solid state.^28^ When the temperature of the solution was elevated from 25 to 65 °C, the absorption spectrum showed no significant changes, indicating the minimized aggregation and π–π stacking of the backbone itself (Fig. S1a†).

On the one hand, the SF-HR film shows intense absorption in the range of 300–550 nm, which is merged with absorption of P3HT from 400 nm to 550 nm and show complementary absorption of PTB7-Th (Fig. S3d†). Unexpectedly, the complementary absorbance in the photoactive layers does not play a role in the photovoltaic properties, which will be discussed in more detail later. The electrochemical properties were estimated by cyclic voltammetry (CV) experiments, as shown in Fig. S1d.† The HOMO and LUMO energy levels of SF-HR were calculated and found to be −5.53 eV and −3.35 eV, respectively. The HOMO energy levels of P3HT and PTB7-Th were determined to be −5.14 eV and −5.23 eV and the LUMO energy levels of P3HT and PTB7-Th were −3.14 eV and −3.63 eV, respectively (Fig. S3†). All optical and electrochemical data pertaining to the SF-HR acceptor are summarized in Table 1. Fig. 1c shows the optimized molecular geometry of SF-HR that is determined by density functional theory (DFT) calculations with the B3LYP 6-31G basis set. As expected, SF-HR is highly twisted between two ringed fluorene units; the dihedral angle between the fluorene ring systems is 69.5°. Meanwhile, as shown in Fig. 1d, the structural order of SF-HR was investigated using grazing incidence wide-angle X-ray diffraction (GIWAXS). It was found that neat SF-HR films show no obvious diffraction peaks, leading the SF-HR acceptor is nearly amorphous. Thus, it can be conducted that the twisted 3D structure creates a 3D charge-transporting network, improving the electron mobility.^24^

**Table 1:** Optical and electrochemical properties of SF-HR

Small molecule	*T*_d_ a [°C]	*λ*_max_ [nm] solution	*λ*_max_ [nm] film	*λ*_onset_ [nm] film	*E*_g_^opt^ b [eV]	*E*_g_^CV^ [eV]	HOMO [eV]	LUMO^opt^^c^ [eV]	LUMO^CV^ [eV]
SF-HR	330	475	478	550	2.20	2.18	−5.53	−3.33	−3.35

a Decomposition temperature (*T*_d_) was determined by TGA (with 5% weight loss).

b Estimated values from the UV-vis absorption edge of the thin films (*E*_g_^opt^ = 1240/*λ*_onset_ eV).

c Calculated from HOMO energy levels and optical band gaps.

### 2.2. Photovoltaic properties

**Fig. 2:**
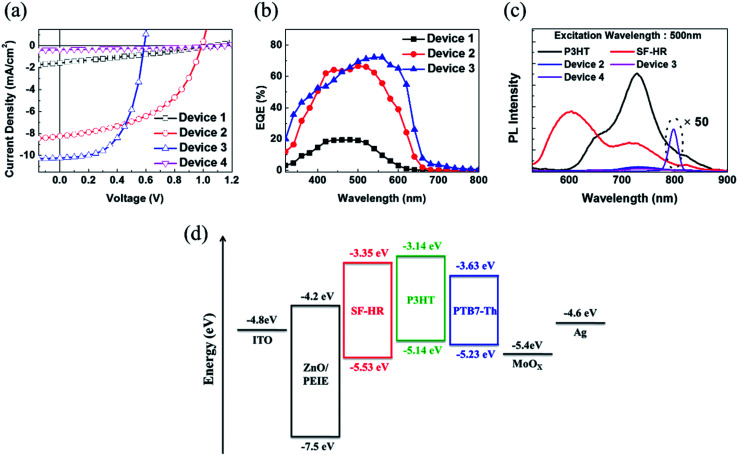
(a) *J*–*V* and (b) EQE curves (c) PL spectrum of device 1 (as-cast P3HT:SF-HR), device 2 (annealed P3HT:SF-HR), device 3 (annealed P3HT:PC71BM), device 4 (annealed PTB7-Th:SF-HR). (d) Energy band diagram of the devices in this study.

Based on the above results, OSCs using the inverted device configuration of ITO/ZnO nanoparticles (NPs)/ethoxylated polyethylenimine (PEIE)/photoactive layer/MoO_*X*_/Ag were fabricated and tested under simulated 100 mW cm^−2^ AM 1.5 G illumination. The photovoltaic results are summarized in Table 2, and the corresponding *J*–*V* curve is illustrated in Fig. 2a. Detailed optimized tests are shown in the ESI (Tables S1–S5, Fig. S5–S8†). Notably, the best photovoltaic performance was estimated with D/A ratios of 1 : 1 and a photoactive layer thickness of approximately 100 nm. OSCs based on as-cast P3HT:SF-HR provide a PCE of 0.46% with a *V*_OC_ value of 1.06 V, a short-circuit current density (*J*_SC_) of 1.54 mA cm^−2^, and a fill factor (FF) of 28%. Upon thermal annealing (TA) at 120 °C for 10 min, a highly enhanced PCE of 4.01% was obtained, with a *V*_OC_ value of 1.00 V, a *J*_SC_ value of 8.23 mA cm^−2^, and a FF value of 49%. This system yielded a significantly improved PCE and even a high *V*_OC_ value of 1 V as compared to PC_71_BM (a PCE of 3.2% with a *V*_OC_ value of 0.57 V, a *J*_SC_ value of 10.35 mA cm^−2^, and a FF value of 55%). Due to the high-lying LUMO energy level of SF-HR (−3.3 eV) and the minimized energy level offset between the P3HT and SF-HR component, high *V*_OC_ values resulted. In contrast, the PTB7-Th:SF-HR device exhibits a poor PCE of 0.13%. Even though PTB7-Th show promising complementary absorption spectrum and HOMO level matching (*V*_OC_ value of 1.15 eV) with SF-HR, the LUMO energy level alignments between PTB7-Th and SF-HR is unsuitable electron transfers. In Fig. 2d, the energy band diagram indicates that the HOMO and LUMO energy level of SF-HR are sufficiently deeper than those of P3HT, but the LUMO is significantly higher than that of PTB7-Th. The offset of the HOMO and LUMO (0.4 eV and 0.2 eV) energy level between SP-HR and P3HT is sufficient to achieve a driving force that encourages exciton dissociation and avoids charge recombinations.^29^ The LUMO level of PTB7-Th, however, is lower than that of SF-HR (∼0.3 eV), possibly leading to inefficient exciton dissociation due to the unmatched energy levels.^30,31^ As a result, a poor PCE of 0.13% with a *V*_OC_ value of 1.15 V, a *J*_SC_ value of 0.36 mA cm^−2^, and a FF value of 31% were estimated. It is clear that the P3HT:SF-HR device exhibits photovoltaic performance superior to that of fullerene-based OSCs, which is mainly attributed to the greatly increased *V*_OC_. This was caused by the perfectly aligned energy levels with a minimized energy level offset. In particular, when compared with a PTB7-Th:SF-HR device, the importance of the perfect agreement with the energy levels between the donor and acceptor is clear. Fig. 2b shows the EQE spectra of the devices from 300 to 700 nm. Without TA, the EQE of the P3HT:SF-HR device reached only 20%, from 400 to 600 nm. In contrast, the device with TA exceeded 60%. Interestingly, the P3HT:PC_71_BM device showed a greater increase in *J*_SC_ (10.22 mA cm^−2^ for P3HT:PC_71_BM *vs.* 8.23 mA cm^−2^ for P3HT:SF-HR). The slightly higher *J*_SC_ can be attributed to its stronger response in the wavelength region of 500–650 nm, allowing a greater contribution to light harvesting and higher charge carrier mobility in photoactive films.

**Table 2:** Photovoltaic properties of P3HT:SF-HR, P3HT:PCBM, PTB7-TH:SF-HR with thermal annealing at 120 °C for 10 min under 1 sun illumination (AM 1.5 G, 100 mW cm^−2^)

Polymer	Acceptor	Annealing [10 min]	*V*_OC_ [V]	*J*_SC_ [mA cm^−2^]	FF [%]	PCE [%]	*μ*_h_ c [cm^2^ V^−1^ s^−1^]	*μ*_e_ d [cm^2^ V^−1^ s^−1^]	Device
P3HT	SF-HR	As-cast	1.06 (1.04 ± 0.02)^b^	1.54 (1.50)^a^ (1.43 ± 0.12)^b^	28 (26 ± 2)^b^	0.46 (0.31 ± 0.16)^b^	1.05 × 10^−5^	6.17 × 10^−7^	1
		120 °C	1.00 (0.99 ± 0.01)^b^	8.23 (8.16)^a^ (8.10 ± 0.14)^b^	49 (46 ± 3)^b^	4.01 (3.82 ± 0.18)^b^	1.02 × 10^−4^	1.65 × 10^−6^	2
	PCBM	120 °C	0.58 (0.56 ± 0.02)^b^	10.22 (10.08)^a^ (10.01 ± 0.22)^b^	56 (54 ± 2)^b^	3.36 (3.14 ± 0.22)^b^	3.11 × 10^−4^	2.73 × 10^−5^	3
PTB7-Th	SF-HR	120 °C	1.15 (1.14 ± 0.02)^b^	0.36 (0.28 ± 0.07)^b^	31 (30 ± 2)^b^	0.13 (0.07 ± 0.06)^b^	—	—	4

a The value is calculated from EQE data.

b The average PCE in the brackets is obtained from over 20 independent devices.

c Hole-only device is ITO/PEDOT:PSS/photoactive layer/Au.

d Electron-only device is ITO/ZnO NPs/PEIE/photoactive layer/Ca/Al.

To understand the charge transport behavior with this combination of the donor and acceptor, a photoluminescence (PL) spectroscopy assessment was carried out (Fig. 2c). The PL spectra present the neat emission spectra of P3HT and SF-HR with emission maxima at 740 nm and 600 nm, respectively. The P3HT:SF-HR blended film shows that the PL emission of SF-HR was successfully quenched by P3HT under excitation at 500 nm, implying that photo-induced excitons can be extracted as electrons at the P3HT and SF-HR surface. In the PL spectra of the PTB7-Th:SF-HR blend film, the PL quenching of SF-HR does not appear and showed strong emission peak at 800 nm due to the energy level mismatching between acceptor and donor polymer, indicating energy transfer from the SF-HR to PTB7-Th. Instead of charge carrier transfer, the energy transfer is possible from SF-HR to PTB7-Th under excitation wavelength at 500 nm, because the emission of acceptor and the absorption of donor polymer is overlapped and only strong emission peak of PTB7-Th is generated at 800 nm.^32,33^

Furthermore, to classify the relationship between the charge carrier mobility and the device performance, we determined the hole and electron mobility from space-charge-limited current (SCLC) measurements of typical photoactive layers, as presented in Fig. S4.† Before TA, the hole (*μ*_h_) and electron (*μ*_e_) mobility of the P3HT:SF-HR blended films is 1.05 × 10^−5^ and 6.17 × 10^−7^ cm^2^ V^−1^ s^−1^, respectively. These two values were correspondingly increased to 1.02 × 10^−4^ and 1.65 × 10^−6^ cm^2^ V^−1^ s^−1^ after TA. This implies that TA can encourage intermolecular interaction in photoactive films, which would be responsible for the improvement of the photovoltaic properties, especially the *J*_SC_ and FF values. For the P3HT:PC_71_BM blended system, *μ*_h_ was estimated to be 3.11 × 10^−4^ cm^2^ V^−1^ s^−1^, similar with that of the TA P3HT:SF-HR film. However, the P3HT:PC_71_BM film showed a relatively high *μ*_e_, 2.73 × 10^−5^ cm^2^ V^−1^ s^−1^, which is approximately one order of magnitude higher than that of the P3HT:SF-HR film. These high electron mobility and balanced charge carrier mobility values are ascribed to the enhanced *J*_SC_ and FF values compared to those of SF-HR-based OSCs. It can be speculated that despite the low electron mobility and *J*_SC_ value of the P3HT:SF-HR film compared to the P3HT:PC_71_BM system, TA can promote an intermolecular network in the P3HT:SF-HR photoactive layers, leading to stronger absorption and better charge transport, which are responsible for the improved photovoltaic properties.

Interestingly, to investigate the intrinsic property of the unipolar electron acceptor characteristics for organic electronic devices, OSCs were fabricated with the inverted structure of ITO/ZnO NPs/PEIE/SF-HR:PC_71_BM/MoO_*X*_/Ag, with a weight ratio of 1 : 1 SF-HR:PC_71_BM. As shown in Fig. S9,† although the energy level was well matched with SF-HR as a donor and PC_71_BM as an acceptor, the photovoltaic results were quite low, indicating the lack of feasibility as a proper OSC device. It is support that SF-HR is outstanding molecule as an n-type small-molecule acceptor.

### 2.3. Charge generation, recombination and extraction

**Fig. 3:**
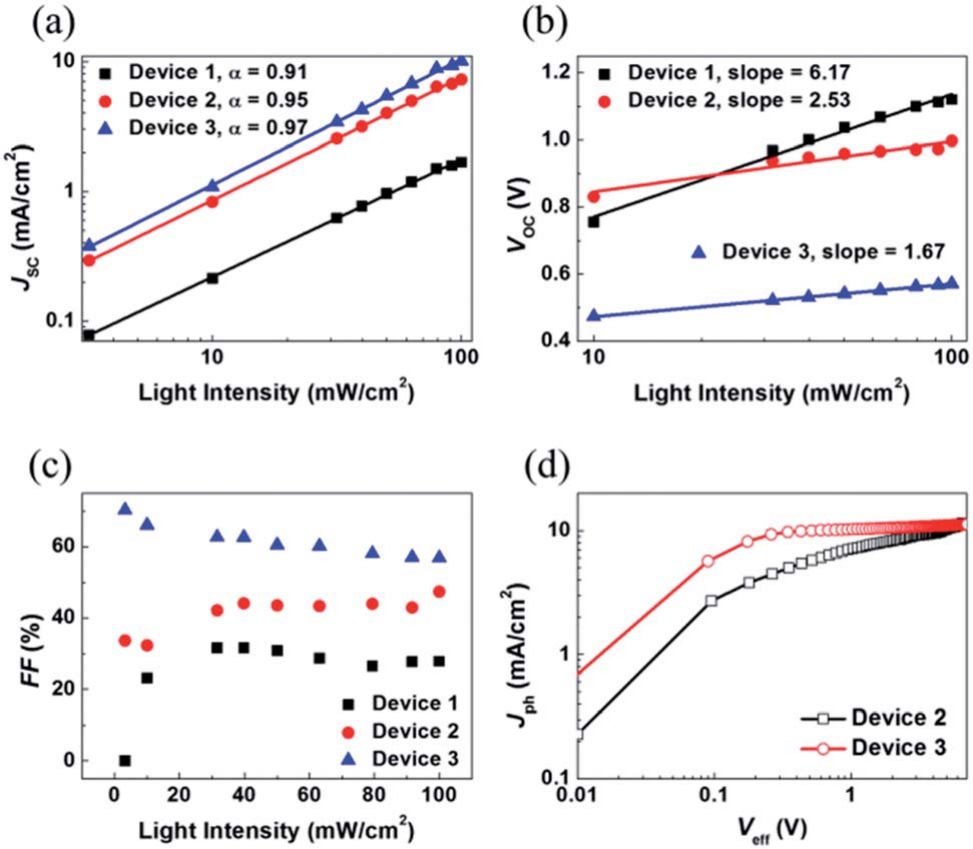
Light intensity dependence of (a) *J*_SC_, (b) *V*_OC_, (c) FF and (d) photocurrent density (*J*_ph_) as a function of the effective voltage (*V*_eff_) for device 1 (P3HT:SF-HR as-cast device), device 2 (P3HT:SF-HR annealed device), device 3 (P3HT:PC_71_BM annealed device).

To gain a better understanding of the relationship between the charge recombination mechanism and the photovoltaic parameters, the dependence of the *J*_SC_, *V*_OC_, and FF characteristics on the intensity of light was examined. The *J*–*V* characteristics of P3HT:SF-HR and P3HT:PC_71_BM OSCs depending on light intensity are shown in Fig. S10.† As shown in Fig. 3a, *J*_SC_ is presented as a function of the light intensity. The curve was fitted according to the power-law dependence of *J*_SC_ on the light intensity. It can be carried out by solving *J*_SC_ ∝ *P*_light_^*α*^, where the value of the power law scaling exponent *α* indicates the strength of the bimolecular recombination. The more *α* approaches unity, the lower the possibility of bimolecular recombinations.^18,19^ Here, a larger *α* (0.95) was obtained with the P3HT:SF-HR device with TA processing than for the device processed without TA (*α* = 0.91), indicating the reduced possibility of bimolecular recombination losses in the TA-processed device, leading to the promotion of higher *J*_SC_ and FF values. Indeed, the relatively large *α* value was estimated to be 0.97 for P3HT:PC_71_BM, suggesting more negligible bimolecular recombinations compared to SF-HR NFA-based OSCs. In other words, it may undergo sufficient charge transfers with less interruption of the photo-induced charge carrier decay, leading to a significantly increased photo-induced current density. On the other hand, Fig. 3b presents a plot of *V*_OC_ as a function of the light intensity based on *V*_OC_ ∝ *nkT*/*q* ln(*P*), where *k* is the Boltzmann constant, *T* is the temperature in kelvin, *q* is the elementary charge and *P* is the light intensity.^34^ For a trap-assisted recombination system, a slope of 2*kT*/*q* is obtained. In contrast, a value close to unity is estimated such that bimolecular recombinations are the main defects of charge carrier recombination losses. Device 1 and device 2 showed a slope of 6.17*kT*/*q* and 2.53*kT*/*q*, respectively, while device 3 attained smaller slope 1.67*kT*/*q*. The deviation from the value (>2*kT*/*q*) indicates that trap-assisted charge recombination takes place in all of these devices. Especially, SF-HR-based OSCs are highly affected by the trap-assisted charge recombination. It might be that a recombination process is generated in 3D-structured acceptor being determined by the trap states regarded as the dominant recombination pathway. After treated thermal annealing, however, the trap sites are complemented due to improved morphology and inter-mixing properties and then the reduction of recombination is happened.^47,48^

Additionally, to investigate the cause of the higher FF in the PC_71_BM device as compared to that in the SF-HR device,^35^ a qualitative study was undertaken of FF as a function of the light intensity (Fig. 3c). For operating close to maximum power, the FF is the significant effective factor. The light intensity dependence of the FF reveals that there is some competition between bimolecular and trap-assisted recombination in OSCs. When supporting the dominance of bimolecular recombination, the value of FF increases with decreasing incident light intensity as the recombination rate is proportional to the product of charge carrier densities. In case of trap-assisted recombination, FF deteriorates with reducing incident light intensity as the proportion of free charges recombining with trapped charges increases.^49,50^ As shown in Fig. 3c, the device 1 (P3HT:SF-HR as-cast device) and device 2 (P3HT:SF-HR annealed device) showed different patterns. At low light intensity (up to 40 mW cm^−2^) for device 1, the FF was increased, and then that is almost retained as around 30%, while the FF of device 2 is gradually increased. For the device 1, the tap-assisted recombination dominated for light intensity above 40 mW cm^−2^. In case of device 2, however, the trap-assisted recombination is significantly effective and dominant under the all light intensity. The FF of device 3 (P3HT:PC_71_BM annealed device) is slightly decreased and then relatively stable, indicating that bimolecular recombination is the main loss channel in this device. And P3HT:PC_71_BM yielded the best FF values at nearly all light intensities, indicating that it was capable of the most efficient charge extraction and transport to the respective electrodes given the fewer charge recombinations. In addition, to gain insight into the effect of P3HT donor incorporation with SF-HR or PC_71_BM as an acceptor on the photocurrent and exciton dissociation with saturation photocurrent density (*J*_sat_) and charge dissociation probabilities *P*(*E*,*T*), the P3HT:SF-HR and P3HT:PC_71_BM devices were assessed. Fig. 3d presents the photocurrent density (*J*_ph_) *versus* the effective voltage (*V*_eff_) curve. *J*_ph_ is defined *via J*_L_ − –*J*_D_, where *J*_L_ and *J*_D_ are the current densities in illumination and darkness conditions, respectively. *V*_eff_ is equal to *V*_0_ − *V*_appl_, where *V*_0_ is the voltage when *J*_ph_ = 0 and *V*_appl_ is the applied bias voltage.^36,37^ For the P3HT:PC_71_BM system, *J*_ph_ shows nearly linear dependence on the voltage, reaching saturation at a low value of *V*_eff_ and suggesting that the photo-induced excitons are extracted into free carriers with greatly effective charge carrier collection as compared to the SF-HR device. Only 68% of the photo-generated excitons were dissociated in the P3HT:SF-HR device, causing a rather low contribution to *J*_SC_, which indicates that geminate charge recombination is a source of major losses. The *P*(*E*,*T*) value was calculated as 83% for P3HT:PC_71_BM leading the lower possibility of geminated recombination losses of excitons in the PC_71_BM device. It is supported by the higher *J*_SC_ and FF values compared with P3HT:SF-HR device.^38^

### 2.4. Morphological characterizations

**Fig. 4:**
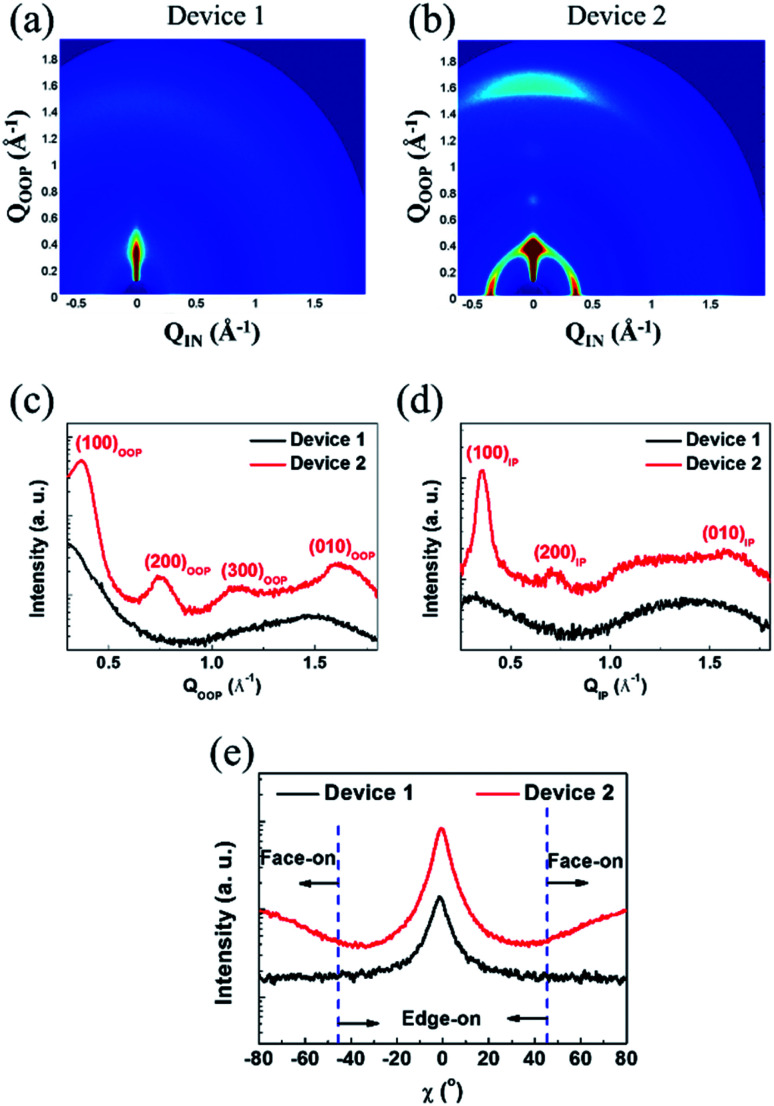
GIWAXS images of (a) device 1 (as-cast P3HT:SF-HR), (b) device 2 (annealed P3HT:SF-HR). Line-cuts profiles from GIWAXS patterns of (c) out-of-plane (OOP), (d) in-plane (IP). (e) Pole figures for device 1 and device 2.

To classify the morphological characteristics of the photoactive layers, we applied GIWAXS, atomic force microscopy (AFM), and transmission electron microscopy (TEM) measurements. The diffraction images and the related in-plane (IP) and out-of-plane (OOP) line-cut profiles are correspondingly displayed in Fig. 4, S11, and S12.† The pure P3HT film showed an aligned molecular orientation, corresponding to a lamellar (*d* = 16.83 Å) and the π–π stacking (*d* = 4.00 Å) diffraction peak of the edge-on orientation with a face-on orientation of lamellar (*d* = 17.90 Å) and π–π stacking (*d* = 3.95 Å) (see Fig. S11 and Table S6†). For the as-cast P3HT:SF-HR films, no clear diffraction peaks appeared, which may be due to the relatively amorphous orientation with a lack of an intermolecular packing system between the donor and acceptor. On the other hand, the thermal annealed P3HT:SF-HR film showed a clear diffraction peak along the IP and OOP directions with (100) diffraction at distances of 17.65 Å and 17.00 Å, respectively. In the OOP direction, the P3HT:SF-HR film with TA also exhibited strong π–π (010) diffraction with a distance of 3.91 Å, corresponding to the face-on oriented P3HT domain.

To compare the pole figures of the annealing systems in the P3HT:SF-HR photoactive films, their (100) lamellar diffraction profiles were extracted (Fig. 4c).^39^ According to a previous report, integrated peak areas with polar angles (*χ*) of 0–45° and 135–180° (*A*_*xy*_) and 55–125° (*A*_*z*_) represent the fractions of the face-on and edge-on crystallites, respectively.^40–42^ Thus, the degree of face-on orientation in P3HT:SF-HR with TA is substantially higher than that of the As-cast BHJ photoactive film, which is beneficial for vertical charge transport. In the P3HT:PC_71_BM blends (see Fig. S12a†), more donor crystallites clearly existed as compared to those in the SF-HR blended film, giving rise to a phase-separated morphology dominated by the edge-on crystallization of P3HT. Furthermore, to understand the molecular packing behavior between PTB7-Th:SF-HR in the photoactive films, the molecular orientation was also investigated. Fig. S11b† revealed that the neat PTB7-Th showed strong face-on crystallites, while the crystallinity of the PTB7-Th donor had collapsed upon blending with the acceptor molecule, SF-HR, which is critical to the charge carrier extraction and traveling, as shown in Fig. S12b.† The detailed *d*-spacing parameters are listed in Table S6.† Consequently, these results demonstrate clearly that a TA treatment can promote the crystallization and preferred face-on stacking of P3HT in SF-HR-based photoactive layers, resulting in much better charge transport capabilities.

**Fig. 5:**
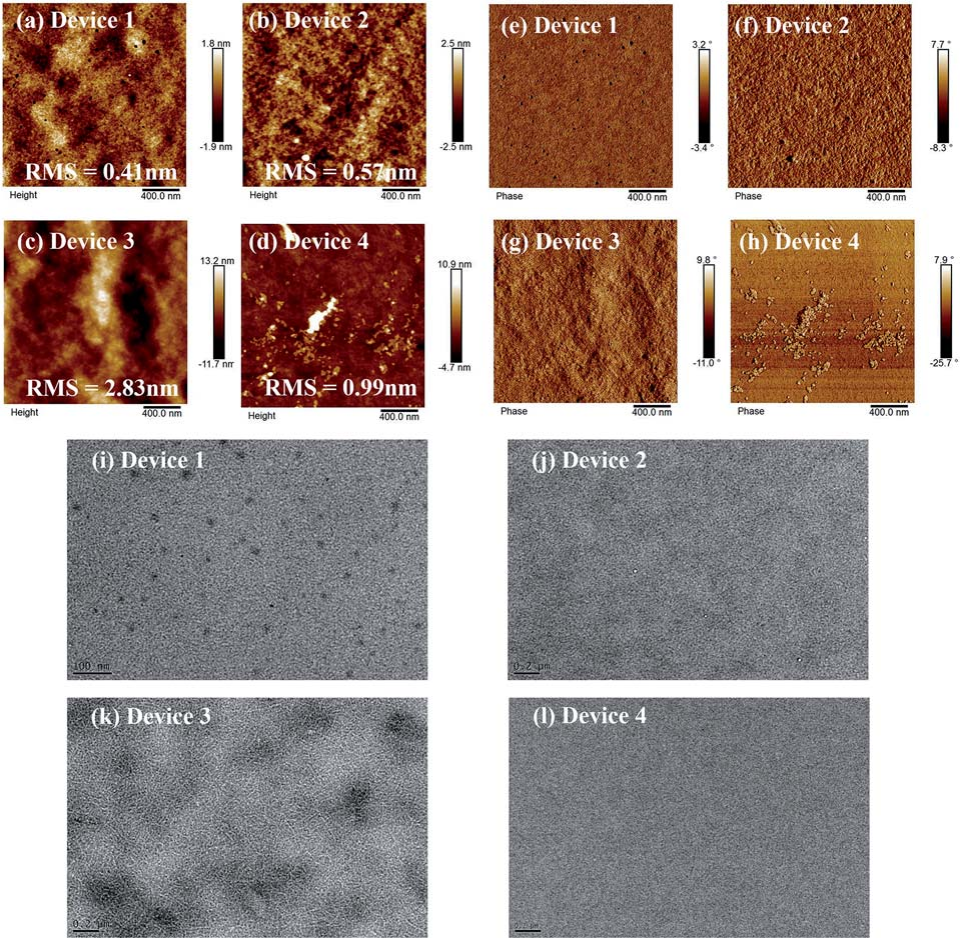
AFM height, phase and TEM image of the (a, e and i) device 1 (as-cast P3HT:SF-HR), (b, f and j) device 2 (annealed P3HT:SF-HR), (c, g and k) device 3 (annealed P3HT:PC_71_BM), (d, h and l) device 4 (annealed PTB7-Th:SF-HR).

Given the above results, the AFM surface images of those films also showed apparent differences. As shown in Fig. 5, images of the TA-treated P3HT:SF-HR BHJ films show a smooth and uniform surface with a root-mean-square (RMS) surface roughness of 0.57 nm. For the blended film of P3HT:PC_71_BM, aggregates with a surface roughness value of 2.83 nm and clearly enhanced nanoscale-phase separation are readily observable. In contrast, the blended film of PTB7-Th:SF-HR exhibited a surface with an irregular morphology and with a RMS value of 0.99 nm.

On the one hand, TEM measurements were carried out to examine the phase separation of the photoactive films (see Fig. 5). The as-cast P3HT:SF-HR film showed no clear fibrous features and an obvious spherical aggregated spot, while the photoactive film with TA showed the clear but small fiber structure of the P3HT polymer. In contrast, the P3HT:PC_71_BM film exhibited a homogeneous morphology with the clear fiber structure of the P3HT polymer, which improved the charge separation and charge carrier transport capabilities. Therefore, the beneficial morphology caused by thermal processing in the P3HT:SF-HR photoactive film promotes the carrier transport pathways and increases *J*_SC_ and FF. The GIWAXS, AFM and TEM images support that P3HT:SF-HR film show the greater morphological structure, resulting in charge extraction and transportation between the donor and acceptor interface. Overall, the evident morphological formation based on the P3HT:SF-HR systems demonstrates the donor aggregated more strongly after TA influencing the impressive enhancement of the photovoltaic performance despite combination with the amorphous acceptor.

### 2.5. Device stability

**Fig. 6:**
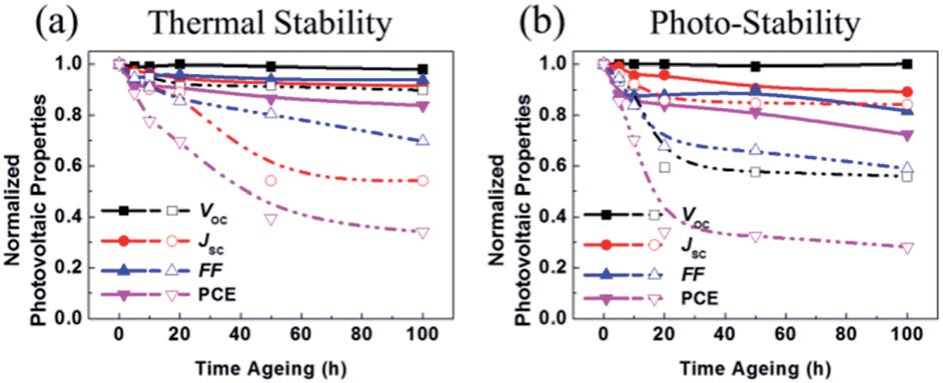
Variation of normalized PCE of the devices based on P3HT:SF-HR (filled), P3HT:PC_71_BM (unfilled) for (a) thermal stability test at 120 °C, for 100 h, (b) light soaking with glass-to-glass encapsulation under the 1 sun illumination.

It should be noted that the stability of OSCs was estimated through thermal and photo degradation tests. First, in order to compare the thermal stability of the P3HT:acceptor blends, photoactive films were heated to 120 °C for 100 h. The corresponding time-discovered photovoltaic properties are shown in Fig. 6a. The P3HT:SF-HR device showed much better thermal stability than the PC_71_BM device, displaying about 83% of its initial PCE even after 100 h. It was observed that the P3HT:PC_71_BM device was degraded under heat stress (see Fig. 6a and Table S7†). The PCE of this device dropped rapidly to a considerably low 34% within 100 h. This outcome indicates that the greatest initial drop in the PC_71_BM-based OSCs under thermal stress was due to the production of its micrometer-scale aggregation,^43,44^ which facilitates the formation of the charge carrier trap state and morphological changes. The trends of the other photovoltaic parameters (*V*_OC_, *J*_SC_, and FF) imitated the PCE trend.

In contrast, it can be suggested that due to the amorphous feature of the SF-HR acceptor, no distinct aggregation can occur. As a result, the PCEs of the P3HT:SF-HR-based OSCs were mostly retained during the thermal treatment.

Furthermore, we investigated the photo-stability of OSCs under continuous exposure to AM 1.5 G, under glass-to-glass encapsulation for 100 h. Fig. 6b and Table S8† show the normalized photovoltaic parameters of the P3HT:SF-HR and P3HT:PC_71_BM OSCs. The PCE of the P3HT:SF-HR device retained ≈72% of its initial value for up to 100 h, while the P3HT:PC_71_BM device retained about 28% of its initial efficiency under identical conditions. The degradation of the device performance is usually caused by decreases in the *V*_OC_ and FF values during illumination. The inferior parameters can be explained by possible light-induced morphological and molecular structural degradation in the PC_71_BM-based photoactive material, which causes a decline in the charge carrier transport.^45,46^ Meanwhile, these phenomenon is in good agreement with the fact that the P3HT:SF-HR film showed superior retained PCEs of its initial results and better morphological stability than P3HT:PC_71_BM under the thermal and photo stress.

## 3. Conclusion

In summary, a spirobifluorene-core-based non-fullerene electron acceptor, SF-HR, is designed and synthesized. Although SF-HR is a highly twisted molecular structure with amorphous characteristics, it exhibits promising potential for application to OSCs. Specifically, SF-HR resembled particularly well the classic p-type polymer P3HT. Owing to the aligned energy level, good intermolecular networking with profound face-on orientation and n-type unipolar behavior, OSCs based on P3HT:SF-HR blended films exhibit the best PCE of 4.01% with an outstandingly high *V*_OC_ of 1.00 V. Moreover, the P3HT:SF-HR-based devices also showed excellent thermal and photo stability capabilities during a thermal treatment at 120 °C and a 1 sun light soaking test lasting 100 h. As compared to the PC_71_BM system, P3HT:SF-HR showed much higher thermal and photo stability results, retaining 83% and 72% of its initial PCEs. These results demonstrate that SF-HR is a promising non-fullerene acceptor suitable for future practical applications in OSCs.

## 4. Experimental section

Fabrication and characterization of the organic solar cells (OSCs): in this study, the devices were fabricated with ITO/ZnO NPs/PEIE/photoactive layer/MoO_*X*_/Ag. The procedure for cleaning the ITO glass substrate included sonication and rinsing in deionized water, isopropyl alcohol, and acetone. A thin layer of ZnO NPs (2.5 wt% ZnO in 1-butanol purchased from Nano Clean Tech, Republic of Korea) was spin coated at 5000 rpm for 30 s to get average thickness of 30 nm onto the UV-ozone treated ITO substrate and then annealed at 100 °C for 10 min in air. And 0.2 wt% PEIE solution was prepared in 2-methoxy ethanol and was spin coated at 4000 rpm for 30 s over the ZnO layer, followed by 100 °C thermal treatment for 10 min. Before use, the ZnO NPs and PEIE solution were filtered through a 0.45 μm filter. For deposition of the photoactive layer, donor : acceptor (1 : 1) dissolved in chloroform with a total concentration 15 mg ml^−1^ were spin-cast at 2000 rpm for 20 s in the N_2_-filled glove box to get thickness around 100 nm on top of the PEIE layer. After photoactive layer deposition, substrates were thermal annealed on hot plate at 120 °C for 10 min. Finally, 10 nm thick MoO_*X*_ and 100 nm Ag were deposited successively to complete the inverted device structure. The photoactive area of the OSCs was 12 mm^2^. OSCs efficiencies were characterized under simulated 100 mW cm^−2^ AM 1.5 G irradiation from a Xe arc lamp with an AM 1.5 global filter. Simulator irradiance was characterized using a calibrated spectrometer, and the illumination intensity was set using an NREL-certified silicon diode with an integrated KG1 optical filter. The EQE was measured by underfilling the device area using a reflective microscope objective to focus the light output from a 100 W xenon lamp outfitted with a monochromator and optical chopper; the photocurrent was measured using a lock-in amplifier, and the absolute photon flux was determined using a calibrated silicon photodiode. All device measurements were carried out in air at room temperature.

## Conflicts of interest

There are no conflicts of interest to declare.

## Supplementary Material
